# Addressing the Ethical, Legal, and Social Issues of Healthtech in Education: Insights From Japan

**DOI:** 10.2196/72781

**Published:** 2025-07-18

**Authors:** Motofumi Sumiya, Tomoko Nishimura, Kyoko Aizaki, Ikue Hirata, Nobuaki Tsukui, Yuko Osuka, Manabu Wakuta, Atsushi Senju

**Affiliations:** 1 Research Center for Child Mental Development Hamamatsu University School of Medicine Shizuoka Japan; 2 United Graduate School of Child Development, Osaka University, Kanazawa University, Hamamatsu University School of Medicine, Chiba University and University of Fukui Osaka Japan; 3 Department of Clinical Psychology Hanazono University Kyoto Japan; 4 Institute of Child Developmental Science Research Shizuoka Japan; 5 Faculty of Global Studies Reitaku University Chiba Japan; 6 Faculty of Education and Integrated Arts and Sciences Waseda University Tokyo Japan

**Keywords:** health technology, ethical, legal, and social issues, education, school, well-being

## Abstract

The increasing application of health technology (healthtech) in educational settings, particularly for monitoring students’ mental health, has garnered significant attention. These technologies, which range from wearable devices to digital mental health screenings, offer new opportunities for enhancing student well-being and strengthening support systems. Numerous studies have explored the ethical, legal, and social issues (ELSIs) of healthtech in the field of psychiatry, highlighting its potential benefits while also acknowledging the inherent complexities and risks that demand careful consideration. However, the ELSIs related to the use of healthtech in educational settings remain largely overlooked and insufficiently addressed. This study provides an overview of items that should be considered by researchers, teachers, and education boards or committees to promote healthtech in the educational context. By adapting existing ELSI frameworks from educational technology and digital health, this study systematically reviews ethical concerns surrounding healthtech in schools. Expert consultations were conducted through a project consisting of members with expertise related to healthtech, including developers, a teacher, a school counselor, and university researchers, leading to the identification of 52 ELSI concerns categorized into 8 domains: consent, rights and privacy, algorithms, information management, evaluation, use, role of public institutions, and relationships with private companies. Using Japan as a case study, we examine regulatory and cultural factors affecting healthtech adoption in schools. The findings reveal critical challenges, such as ensuring informed consent for minors, protecting student privacy, preventing biased algorithmic decision-making, and maintaining transparency in data management. In addition, institutional factors, including the role of public education policies and private-sector involvement, shape the ethical landscape of healthtech implementation. This study highlights the need for multistakeholder collaboration to establish guidelines that balance innovation with ethical responsibility. The study underscores the need for a multifaceted approach to mitigate risks such as data misuse, inequitable access, and algorithmic bias, ensuring the ethical and effective use of healthtech in education. The fundamental ELSI framework for healthtech, including privacy, consent, and algorithms, can be applied to educational systems worldwide, while aspects related to public education policies should be considered in accordance with the specific context of each country and culture. Incorporating healthtech into the educational system helps address the barriers associated with traditional approaches, including limited resources, cost constraints, and logistical challenges. Researchers from universities and healthtech companies, along with educators and other stakeholders, should ensure that healthtech projects consider diverse ELSI concerns at every stage before and during implementation.

## Introduction

### Overview

The integration of health technology (healthtech) into school education has introduced new opportunities for monitoring students’ mental health and enhancing their well-being. Innovations such as wearable devices that track physical activity (see Au et al [1] and Zhang et al [2] for a systematic review) and daily self-reported mental health screening, provide the potential to revolutionize the educational landscape [3,4]. However, these advancements also raise ethical, legal, and social issues (ELSIs) that require careful consideration. For example, it is imperative to address concerns regarding data privacy, equitable access to healthtech services, and the potential misuse and mishandling of sensitive health information when introducing these technologies in public educational settings. Addressing these concerns ensures that healthtech functions as a supportive tool for students, educators, and parents, rather than a source of harm or inequity. Although many studies have considered ELSIs for healthtech in the field of psychiatry, no study has specifically examined them in the educational context. Accordingly, this study provides an overview of items that should be considered by researchers, teachers, and education boards or committees to promote healthtech in the educational context.

### Importance of Healthtech in Education

There is an increasing emphasis on the role of mental health screening in school education in bridging the gap between the significant mental health needs of students and the limited access to appropriate care. According to Soneson et al [5], identifying mental health challenges in school-aged children early can significantly enhance access to timely interventions and address the widespread issue of underidentification, where only a small percentage of affected children receive adequate support. Similarly, Weist et al [6] emphasized that systematic screening in schools not only enhances outreach to at-risk youth but also integrates mental health initiatives into the educational environment, fostering both mental well-being and academic progress. Effective implementation ensures that such programs align with public health approaches, facilitating early detection and prevention while reducing barriers to learning and combating the stigma surrounding mental health issues.

With the rapid development of digital technology in recent years and its increasing use during the COVID-19 pandemic, mental health screening in schools has become progressively digitalized. The incorporation of healthtech into school-based mental health screening presents a promising solution for addressing the barriers associated with traditional approaches, including limited resources, cost constraints, and logistical challenges. For example, Nishimura et al [3] highlighted the use of tablet devices and health-monitoring apps to examine and analyze students’ daily health and emotional well-being through self-report measures in primary and junior high schools in Japan. They emphasized that the digitalization of data enabled the efficient transition from traditional paper-based data to an online system, facilitating the analysis of both long-term health trends and short-term variations. They also observed that using simple algorithms, such as rolling *z* scores calculated from the 7-day rolling average and SD, to alert students about their health status enabled the rapid identification of data fluctuations. This, in turn, facilitated the early prediction of mental health issues and the timely provision of interventions. These efforts were supported by Japan’s Global and Innovation Gateway for All School Program, which equips students with tablet devices. The introduction of digital technology into school education also has the potential to reduce the burden on teachers, enhance the efficiency and sustainability of observations, and ensure equitable and individually optimized learning environments.

With regard to leveraging healthtech for school-based mental health screening, Porter et al [4] examined the effectiveness of the Digital Health Contact (DHC), an online self-report tool used in schools in the East Midlands of England. Using a structured questionnaire, this tool enables students to provide data on their health and well-being, including indicators of mental health concerns. They introduced a “red flag” system that automatically flags certain answers or keywords when they are included in free-text responses to highlight potential issues using an automated algorithm, directing at-risk students for further evaluation by public health nurses. Porter et al [4] also determined that the DHC enhanced the ability to identify unmet mental health needs among adolescents, particularly in schools with a higher number of students from diverse linguistic backgrounds, where traditional methods often fell short. In addition, while the study noted an increase in the overall number of referrals, it emphasized that the DHC complemented traditional referral pathways, enabling a more structured and proactive approach to providing mental health support. Therefore, although these two studies [3,4] have different objectives and detection algorithms, both further highlight the transformative potential of healthtech in schools, with Nishimura et al [3] focusing on identifying quantitative variation in data and Porter et al [4] emphasizing mechanisms for immediate identification of specific student problems and providing direct support.

However, several issues must be considered when using innovative and novel tools in the context of institutional barriers and traditional rules. Woodrow et al [7,8] highlighted both the promise and challenges of implementing healthtech, such as the DHC in school settings, by conducting qualitative studies with semistructured interviews. From the students’ perspective, the DHC fosters honest communication regarding health and well-being by ensuring privacy and reducing the stigma associated with seeking support [8]. Similarly, stakeholders such as commissioners, service providers, and health care staff emphasized the tool’s effectiveness in identifying unmet health needs and its ability to operate efficiently in resource-limited environments [7]. Importantly, both studies highlight critical ELSI concerns that must be addressed to ensure their responsible use. Concerns regarding data privacy, informed consent, equitable access, and cultural adaptability have been identified as key challenges [7,8]. These findings reinforce the need to introduce robust ELSI frameworks to navigate healthtech in educational settings, ensuring that they promote inclusivity, trust, and ethical practices while maximizing potential benefits.

## Discussion of ELSIs in Healthtech

### Overview

To the best of our knowledge, ELSIs surrounding the use of healthtech in educational contexts have not been systematically evaluated in the literature, although they have been widely explored in the context of psychiatry or mental health informatics [9-14]. These studies have not only highlighted the potential benefits of healthtech but also flagged the inherent complexities and risks that require careful navigation. For example, Shen et al [12] proposed an ethics checklist for deep phenotyping research in psychiatry, combining extensive data collection (such as 24/7 data on location, movement, and emails, along with brain scans, genetics, genomics, neuropsychological assessments, and clinical interviews) with advanced techniques for analyzing these data. The checklist addresses key domains such as informed consent, equity, and privacy, urging researchers to explicitly document their decisions and consult diverse stakeholders. This procedural rigor is crucial in ensuring that healthtech is designed to uphold participants’ rights while addressing systemic inequities stemming from unequal access to digital health solutions. Therefore, by considering a comprehensive ELSI framework based on these perspectives and prioritizing transparency, inclusivity, and accountability at every stage of technology development and deployment, healthtech can advance mental health care and serve as a model for ethically responsible innovations.

Seiferth et al [15] provide a comprehensive set of international guidelines for the development, implementation, and evaluation of healthtech. They emphasize a user-centered and participatory design approach, ensuring that digital solutions address the specific needs of their target populations while enhancing accessibility and usability. The authors identified key considerations including data security, privacy, and transparency, highlighting the importance of building trust among users through clear communication and adherence to ethical standards. They argue that safeguarding user data is critical for maintaining trust, particularly given the sensitive nature of mental health information. The guidelines recommend using advanced security measures such as end-to-end encryption, pseudonymization, and regular security audits to protect against unauthorized access and data breaches. Privacy considerations extend to the ethical collection, storage, and use of personal data. They advocate transparent data practices, including clear communication with users regarding how their data will be managed, who will have access to them, and for what purposes. This transparency must be embedded in the informed consent process to enable users to make informed decisions about their participation in healthtech interventions. In addition, the authors highlight the need for real-time user notifications and accessible options for participants to withdraw their consent or modify their data-sharing preferences. These practices ensure compliance with data protection regulations, such as the General Data Protection Regulation, while fostering a culture of accountability and respect for user autonomy. By addressing these considerations, Seiferth et al [15] provide guidelines aimed at creating a trustworthy environment that encourages the adoption and sustainable use of healthtech for mental health.

### First Step Toward Discussing ELSIs With Regard to the Use of Healthtech in the Educational Context

Although healthtech has penetrated the field of education globally, ELSIs surrounding the use of these technologies have received limited attention. Researchers from universities and healthtech companies, educators, and stakeholders ensure that healthtech projects consider diverse ELSI concerns at every stage before and during implementation. When using healthtech in the educational context, diverse factors must be considered compared to its use in other settings. For example, both students and their parents must be considered when obtaining consent for healthtech use. In addition, because school systems, regulations, and laws related to schools vary by country, it is necessary to consider realistic use within the existing framework of the country where the school is located. For example, in the Japanese education system, with a few exceptions, teaching curricula and assessments are required to adhere to the Courses of Study established by the Ministry of Education, Culture, Sports, Science and Technology (MEXT) [16]. Accordingly, in Japan, it can be challenging for local governments or individual schools to flexibly modify educational content or instructional hours or introduce new technologies and systems. These issues are specific to educational settings and need to be considered, in addition to ELSI concerns related to healthtech for mental health or psychiatry. However, as noted in previous paragraphs, healthtech is increasingly being integrated into the field of education worldwide, yet ELSI considerations regarding the use of these technologies have been largely overlooked.

**Figure 1:**
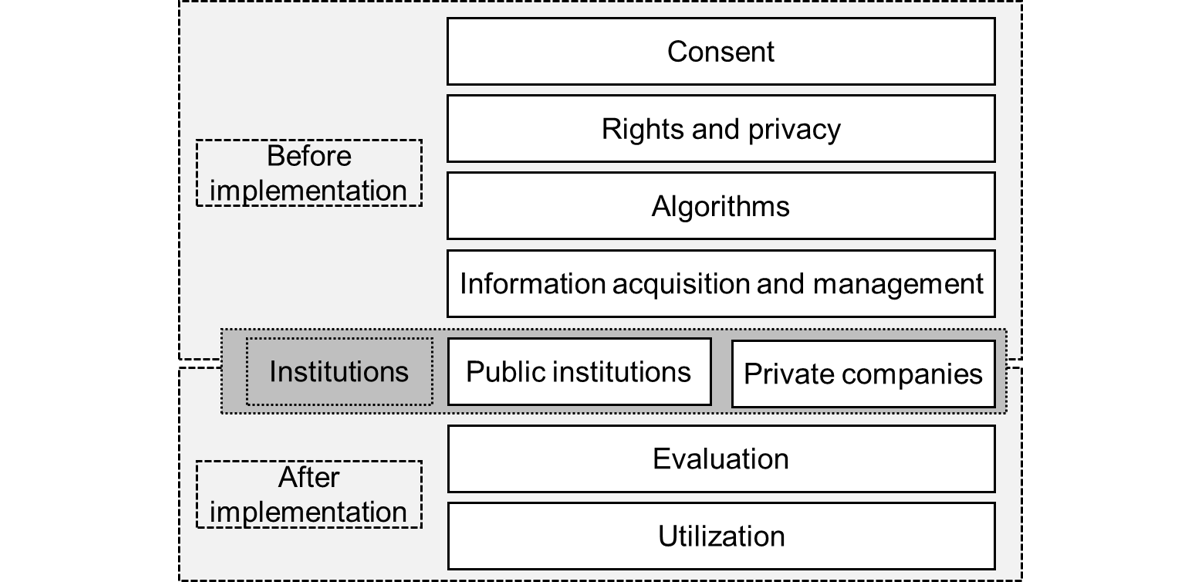
Phased considerations and institutional roles in health technology implementation.

This study examines the issues to be considered when implementing healthtech in Japanese educational settings. Given the need to thoroughly consider the characteristics of Japanese educational settings, we reference a project that examined the ELSIs of implementing educational technology in Japan [17]. Kano et al [17] discuss issues related to the use of digital technology in education, particularly within the context of Japanese-style public education. Their study, conducted through a project involving experts in ELSIs—such as ethicists, legal and constitutional scholars, social psychologists, and digital technology developers—explores 101 ELSI concerns. Our project is supported by the Japan Science and Technology Agency–Mirai Program under the “Society Optimized for Diversity” mission area. This study was conducted by a team comprising healthtech developers, a teacher, a school counselor, and university researchers, with the goal of creating a comprehensive ELSI framework for healthtech in school education. The study design was approved by the ethics committees of Hamamatsu University School of Medicine. To achieve this, we first analyzed the 101 ELSI concerns identified in educational technology research [17]. Each concern was systematically reviewed and assessed for its relevance in healthtech. We conducted a focus group discussion following a structured procedure: individuals with relatively homogeneous attributes, knowledge, and characteristics relevant to the research topic were selected. A moderator facilitated the discussion based on a prepared guideline, encouraging interactions among participants and eliciting their emotions, attitudes, and thoughts on the designated themes. On the basis of participants’ collective input, items from EdTech101 that were not relevant to healthtech were eliminated, while those with strong relevance were grouped and refined. Keywords were assigned to each item, forming the basis for organizing the concerns into 8 thematic categories. This process was grounded in thematic analysis [18,19]. Therefore, we identified 52 key issues, categorized into 8 topics related to healthtech (Table 1; a detailed version is provided in Multimedia Appendix 1, along with the Japanese-language original version). The topics range across (1) consent, (2) rights and privacy, (3) algorithms, (4) information acquisition and management, (5) evaluation, (6) use, (7) public institutions, and (8) private companies (Figure 1), with each containing 4 to 11 issues. In the following section, we discuss the characteristics of each topic and the ELSIs for each.

**Table 1:** Ethical, legal, and social issues on health technology (healthtech) in the educational context.

Category and issues (when data collection and mental health assessments are conducted as a school activity)		Key laws^a^
**Consent**		
	Is guardian informed consent^b^ required? If so, is the informed assent^c^ of students also obtained?	1
	Does the information used in informed consent include risk assessment^d^?	1
	Even in the case where a guardian has not consented to data acquisition from a healthtech service, are students notified of the details of guardian consent/non-consent?	1
	Should an “opt-out^e^” format be applied for consent?	1
	Do students view the facial features and attitudes of teachers and urge their guardians to give their consent, or do they express support?	1
**Rights and privacy**		
	Is there recognition of a right not to be evaluated by healthtech?	1
	To whom, to what extent, and how are the evaluation and reasons disclosed.	1
	Is the so-called right to be forgotten^f^ (e.g., the deletion of information collected by healthtech) recognized?	1 and 2
	Do inferences regarding the internal mind (attitudes, emotions, etc.) of students constitute a failure to protect the freedom of thought and conscience of the concerned persons?	1
	Is this not considered an infringement of the teacher’s right to “educational freedom^g^”?	3
	Does using expression or voice recognition to record the details of student information entail making sensitive information available to others, with a risk of violating privacy rights?	2
**Algorithms**		
	Is third-party oversight (auditing, inspection) guaranteed for algorithms^h^?	1 and 4
	Do mechanisms exist for corrections where, due to inaccurate profiling, mistakes have occurred in the evaluation process?	1
	Can the system consider the need to ensure that students who deviate from the norm are not assessed unfavorably?	1
	Is mental health expertise or third-party certification system required to develop assessment items and algorithms of healthtech? If so, what are the criteria for determining it?	5
**Information acquisition and management**		
	Should sensitive personal information^i^ about students with developmental disabilities and mental health history be acquired and used to screen and sort schools?	1
	Is the information management system regarding information accumulation and management as well as its linking with other information sufficient?	2
	Who, where, and how long should the information collected by healthtech be stored?	—^j^
	How should requests for the cessation of use^k^, etc., of collected data be treated?	6
	Will not schools and teachers emulate the healthtech methods to voluntarily collect excessive personal data?	6
**Evaluation**		
	When an evaluation includes parameters other than just healthtech assessment, how can it be guaranteed that factors indubitably related to prejudice/discrimination do not influence the evaluation?	1
	Does making health/mental condition “visible” promote hierarchization of students by grades?	3
	Is there no danger of so-called overmeasurement from quantifying things that are difficult to quantify?	3
	Is there a risk that visualizing mental health assessments provides a biased view of students? If visualization (with charts, etc.) focuses on mental health assessments rather than school life, is it not possible that more importance will be given to students’ abilities, leading to an over-bias on the importance of a person’s background?	2
	If such a face recognition system encourages a majority of students to adopt expression methods and emotional expressions that tend to result in good score, does this not hinder the diversity of self-expression?	7
	How should one consider and treat cases where sensitive individual information is used to infer, via profiling, that the concerned individual requires special care or consideration?	6
	What are the grounds for making all problems identical for everyone? Should items and assessment methods not reflect regional or age differences?	—
	The use of technologies is expected to increase equality in evaluation of interests, motivation, and attitudes compared with the subjective evaluation of human teachers. However, will objective quantification (scoring) of such things as developmental disorders and/or mental illness result in such conditions being treated in a fixed, inflexible way?	—
	With the accumulation of recordings of a student’s past problematic behavior, even if that student shows growth and development (maturation) over time, will that student not be “stuck with” that reputation, etc., making appropriate evaluations in the future unlikely?	—
	Can mental health history and abilities be linked to personality evaluation?	—
	When the “optimal” value for “individual optimization^l^” recommended by a system is not proven with evidence, is there not a risk that the “individual optimization” as designated by the system in its first introduction and use becomes a type of de facto standard^m^, locking in those values as “standards?”	—
**Use**		
	Are there assurances that the results of evaluations performed to promote the development of a student are not used in selection screening?	1
	It is acceptable if healthtech is used by a teacher as a support tool, but if its influence surpasses that of human judgment, would that lead to “inappropriate control” by HealthTech?	3
	Is healthtech truly used for educational purposes, and not just nominally so?	8
	Will classifications (for determining members of specific school classes, etc.) based on differences in assessment by HealthTech ultimately not lead to discrimination?	9
	If students' posture and attitude are judged based on recognition accuracy (i.e., some faces tend to be recognized by face recognition as actively claiming and others as expressionless), does this constitute discrimination?	9
	Should educational results from healthtech be recognized as the educational performance of teachers?	10
	Is it appropriate to use data obtained through facial recognition^n^, such as a teacher's level of concentration and facial expressions, as criteria for teacher evaluation?	10
	How should the usage of anonymously processed information^o^ be treated?	6
	Is it necessary to add healthtech functions that prioritize student guidance elements, such as those that help improve postures or those that boost students’ self-worth by encouraging them to modify their behavior?	—
	Will the teacher’s duties not be taken over in those areas where healthtech can serve as a substitute or alternate performer?	—
**Public institutions**		
	If there is evidence of educational benefits, is the national government obliged to introduce an educational environment using healthtech?	11
	In the case of digital terminals (tablets, LAN extension inside the student’s home etc.), will the cost of purchasing related educational materials be privately or publicly funded?	11
	If, for example, a system is established where subsidies and other payment standards vary depending on whether or not a certain school has introduced healthtech promoted by the Ministry of Education, Culture, Sports, Science and Technology, could this lead to the penetration of the government's message to children via public education?	3
	Can assistance monies be paid to an educational institution operated by an healthtech enterprise (i.e., an enterprise not designated under article 1 of the School Education Act^p,q^)?	8
	Is it appropriate for the national government to set uniform evaluation standards?	7
	Does the mass introduction of healthtech services entail expenditures of public monies for private businesses?	4
	Does using healthtech to teach mental health count as health education in school curricula?	4
**Private companies**		
	Is it suitable for healthtech provided by private enterprises to be incorporated into educational contents and/or methods?	3
	If healthtech is introduced without sufficient knowledge of ICT^r^, schools may be unable to fully understand its content and ongoing changes, with the result that they merely follow what the tech provider instructs.	8
	Will the value systems and expressions of private companies be linked with the evaluation standards for children/students that are used in public education?	2 and 7
	If a stockholding company, not an incorporated educational institution, possesses multifaceted data of students, is there a risk that the data may be used beyond education-related purposes?	12

^a^Key laws (relevant laws and regulations); 1: Constitution, Article 26, Paragraph 1: “All people shall have the right to receive an equal education correspondent to their ability,” 2: Constitution, Article 13: “Personal rights” and “Right to privacy,” 3: Basic Act on Education, Article 16: “Education must not be subject to improper controls,” 4: Characteristics of Japanese-style public education not necessarily grounded in law, 5: Article 3 of the Educational Personnel Certification Act: 'Educational personnel must be those who have been granted a certificate in accordance with the provisions of this Act, 6: Act on the Protection of Personal Information, 7: Basic Act on Education, Article 1: “Education must be provided with the aim of fully developing the individual character,” 8: Constitution, Article 89: “Expenditures of public money, and limits on its usage (appropriation),” 9: Constitution, Article 14: “Equality under the law” and “Prohibition of discrimination,” 10: Every Article and item (paragraph) of the Local Public Service Act stipulated by this Act,” 11: Constitution, Article 26, Paragraph 1: “Right to receive education “Paragraph 2: “Compulsory education shall be free,” 12: School Education Act, Article 2: “Schools shall be established only by the national government, local governments, and school corporations specified by Article 3 of the Private Schools Act.”

^b^Informed consent: providing consent after having been fully informed and satisfied about the purpose, risks, benefits, and alternatives of the data collection or intervention.

^c^Informed assent: In addition to parental consent (informed consent) for minors and others, the child’s consent should be obtained after he or she understands and accepts the purpose, risks, benefits, and alternatives to data collection or intervention.

^d^Risk assessment: Assessing potential risks and harms that may arise from implementing HealthTech in advance, and considering measures to mitigate those risks.

^e^Opt-out: A process in which individuals, in a situation where they are automatically included in a specific service or data processing, request to be excluded based on personal decision.

^f^Right to be forgotten: The right of an individual to request the deletion of their personal information stored by an organization or service provider when they ask for it to be erased.

^g^Freedom of educational policy: Schools and teachers have the right to freely decide on their educational policy. A famous court case is the Asahikawa School Achievement Test. The “Asahikawa Gakute Judgment” in the 1950s is a leading case involving the freedom of education of school teachers (here, a teacher who opposed the National Achievement Test [or “Gakute”] was charged with obstructing the execution of public affairs).

^h^Algorithm: A set of specific methods, processes, or rules established to address a particular issue or conduct analysis.

^i^Sensitive personal information: Personal information that requires particularly careful handling, such as health status, disability, nationality, criminal history, or beliefs.

^j^Not applicable.

^k^Request for suspension of use: The right of an individual to request the suspension or deletion of personal information when its use is illegal or inappropriate.

^l^Individual optimization: Providing optimal measures and resources based on an individual's needs and characteristics.

^m^De facto standards: Technologies or norms recognized as the default through widespread adoption and proven track record, without undergoing formal standardization.

^n^Face recognition: Analyzing facial features and using it to identify the individual.

^o^Anonymously Processed Information: Data generated by removing or replacing necessary identifying elements from personal information to ensure that specific individuals cannot be identified.

^p^Schools falling under School Education Act 1: Educational institutions (called “schools”) established under article 1 of the “School Education Act.” Specifically, this refers to kindergarten, elementary, junior high, compulsory education, high, secondary education, and special needs education schools, universities, and college of technology (KOSEN).

^q^Schools that do not fall under School Education Act 1: Educational institutions not positioned under article 1 of the “School Education Act.” Specifically, they are international, driving, and preparatory schools.

^r^ICT (information and communication technology): This term collectively refers to technologies and tools related to information processing and communication, such as computers, the Internet, smartphones, and cloud services.

### Consent

The consent obtained by the implementer of healthtech from the participants is an important topic that anyone involved in healthtech needs to address. Shen et al [12] emphasized the importance of obtaining consent after providing participants with sufficient information. Furthermore, when implementing healthtech in educational settings, it is essential to consider who should provide consent for participation and how it should be obtained. First, obtaining informed consent from parents respects their responsibility toward minors and strengthens their trust in the implementation of healthtech. Through this process, parents gain an opportunity to understand the mechanism, purpose, and risks surrounding healthtech, which can provide them with a sense of security. In addition, seeking informed consent from students allows their opinions and autonomy to be respected while fostering an understanding of the importance of decision-making. However, parents’ lack of understanding or low technical literacy may hinder a suitable consent process. For instance, if consent forms contain excessive technical jargon, parents may struggle to comprehend the content and potentially reject participation. Similarly, when obtaining students’ assent, their ability to fully understand the mechanisms and risks of technology depends on their age and developmental stage. Providing appropriate explanations and support is indispensable. If these efforts are insufficient, there is a risk that consent and assent in the school setting will become merely procedural, failing to achieve their intended purposes (Table 1 provides relevant concerns).

Another critical consideration is managing situations where parents withhold consent for healthtech-based data collection. Should students still have access to healthtech, and if so, should they be informed about their parents’ consent decisions? In public education, allowing students to access healthtech even when parental consent has been withheld may be regarded as a student-centered approach that prioritizes their best interests. For instance, in school health management programs providing psychological support or screenings, it may be essential to ensure that students do not miss necessary assistance due to a lack of parental consent. Furthermore, it is crucial to avoid scenarios where specific students are excluded from participation, as this could exacerbate feelings of isolation or stigma associated with healthtech use. Conversely, disregarding parental nonconsent and permitting students to use healthtech could undermine familial trust. Whether or not to inform students about their parents’ decision requires careful deliberation. Informing students could trigger conflicts or resistance toward their parents, while withholding such information could invite criticism for the lack of transparency or ethical oversight in educational settings.

In light of these issues, opt-out systems could be considered an alternative approach to consent, especially in public schools, where initiatives often involve a large number of students. An opt-out approach assumes consent unless parents or students explicitly indicate their objections, which offers several advantages. For example, it can eliminate the need to collect explicit consent from students and their parents, significantly reducing the time and effort required. By including every student unless they actively opt out, the approach increases overall participation and enhances the comprehensiveness of the collected data [20,21]. This facilitates an accurate understanding of the school’s overall situation, which can inform both individual support and broader policy improvements. Furthermore, because participation is considered the default, opt-out systems can help reduce the social stigma often associated with seeking psychological support, thereby improving access to mental health and welfare services.

In Japan, to address these challenges, public schools are required to operate in accordance with established educational regulations, including the School Education Act and the Act on the Protection of Personal Information. When implementing healthtech, schools must ensure proper management based on the guidelines set by the Board of Education and the MEXT. Under the School Health and Safety Act, schools are responsible for ensuring the well-being of students, which includes providing these health-related interventions. For screening related to physical health, it is clearly stated that an explanation to parents and students and parental consent is required if additional medical screening items are added or if privacy-related content is included. However, there is no clear provision for obtaining consent in the implementation of healthtech. This is an ongoing issue for consideration, including opt-out methods.

### Rights and Privacy

Rights and privacy are critical for effective healthtech implementation. In the field of psychiatry, Wies et al [11] suggested that concerns regarding privacy breaches may erode user trust in healthtech, leading to reluctance in sharing data, ultimately diminishing treatment effectiveness. Similarly, in the context of healthtech use in educational settings, it is essential to prioritize the rights and privacy of students. Self-determination, privacy protection, and data management rights are critical elements to safeguard the healthy development and safety of students (Table 1).

First, when implementing healthtech, it is crucial to examine whether students should be granted the right to refuse to undergo mental health assessment. Granting students the right to opt out of evaluations is ethically significant in terms of respecting their self-determination and protecting their privacy. This is particularly important when screening for mental health issues or psychological stress, as students may not feel comfortable disclosing their feelings and conditions. Despite these concerns, forcing evaluations can result in psychological distress. However, broadly recognizing the right to refuse evaluation carries the risk of neglecting issues that require intervention, leaving problems unresolved. Therefore, when introducing healthtech to students, it is necessary to establish clear procedures and alternatives for cases where a student refuses evaluation, ensuring that their decision does not result in disadvantages or inequity.

Next, if healthtech includes the capability to detect sensitive information through nonverbal cues, such as facial expressions or voice, careful consideration is required to avoid potential privacy infringements. For instance, even if students do not intend to disclose their emotions or internal struggles, the system may inadvertently collect and analyze such information covertly. To mitigate this risk, the scope and purpose of data collection must be clearly defined, and comprehensive explanations must be provided to both students and their parents. Furthermore, it is imperative to provide opt-out options during the consent process, allowing participants to decline sharing sensitive information. In cases where interventions or actions are based on information identified by the system, transparency is crucial, as is the implementation of mechanisms that enable both students and parents to review and understand the collected data and subsequent actions.

Finally, it is essential to consider whether the “right to be forgotten”—allowing for the deletion of data collected by healthtech—should be recognized. Data collected in educational contexts, particularly concerning mental health or psychological traits, may include sensitive information. Considering the potential risk such information may pose in the future, a system enabling students or their parents to request data deletion is necessary. However, exercising the right to be forgotten can disrupt the consistency of school health programs or learning support initiatives. Consequently, establishing a framework that minimizes these disruptions when responding to deletion requests is essential. For instance, anonymizing the requested data for deletion and restricting their use in statistical analyses without personal identifiers can be a viable approach. Moreover, it is crucial to clearly outline policies and procedures for data deletion to ensure students, and their parents are informed in advance about the process, its implications, and how deletion requests will be managed.

### Algorithms

The guidelines for developing healthtech in the field of psychiatry emphasize the importance of a user-centered design that actively involves end users, health professionals, and other stakeholders in the preliminary stages of development [15]. This also applies to the field of education, where the design and implementation of algorithms must involve diverse stakeholders and be carefully considered from multiple perspectives. First, ensuring the auditability of algorithms by third parties is critical for maintaining transparency and trust in the system. Healthtech tools implemented in educational settings have a direct impact on how students’ health and learning outcomes are evaluated. If algorithms lack transparency, there is a risk of being unfair or biased. For instance, if an algorithm operates as a “black box,” stakeholders may not understand its decision-making criteria, making it challenging to address unjust evaluations. To mitigate this, mechanisms allowing third-party verification of the algorithm design and decision-making processes should be established. In addition, explaining the auditing process in an accessible manner to schools and families can help build trust among parents and students.

Next, it is important to establish a mechanism to evaluate the accuracy of the assessments derived from the algorithms, as well as a system to correct them if they are found to be inaccurate. There is always a possibility of errors owing to the quality of the input data or system bugs. Therefore, there should be processes that allow students, parents, and teachers to challenge the evaluation results and ensure prompt corrections. For example, when inaccurate results are detected, a system that enables experts to intervene appropriately can protect students’ rights while enhancing the system’s reliability.

Finally, it is important to ensure that algorithms based on standard datasets do not evaluate students with unique characteristics. For instance, students who require special support or come from diverse cultural and social backgrounds may face the risk of unfair treatment based on algorithm-based evaluations. To address this, it is imperative to design algorithms incorporating fairness into their framework. Organizing datasets that account for diverse backgrounds and implementing measures to mitigate harmful biases are critical. Furthermore, combining quantitative algorithmic results with qualitative assessments by teachers and experts can lead to comprehensive and equitable evaluations. By addressing these concerns, healthtech systems can better support students’ diverse needs while ensuring fairness, transparency, and trustworthiness in educational settings (Table 1).

### Information Acquisition and Management

It is essential to carefully consider the ELSI concerns related to the collection and management of information through healthtech [11,12,15]. In an educational setting, the acquisition of sensitive personal data on developmental disorders or mental health conditions requires thorough deliberation. While such information is important for enhancing algorithms’ predictive capabilities related to health conditions, considering its highly sensitive nature, even with student or parental consent, the purposes and methods of its use must be clearly defined. Specifically, if such information is used for class organization or decision-making in educational policies, it is crucial to eliminate any possibility of unfair discrimination or inequitable treatment. To this end, it is necessary to obtain explicit consent from individuals or their parents and ensure that the information collected is used exclusively for educational support. Moreover, a strict privacy protection framework should be established to ensure that sensitive information is not disclosed to other students or teachers.

When institutions beyond schools and boards of education, such as university researchers or private companies, are involved in the design and operation of healthtech, data handling becomes increasingly complicated, necessitating stricter management systems. First, the retention period for this information must be clearly defined. If it is excessively lengthy, the risk of data being used in unforeseen ways increases. For example, data related to a student’s health condition or psychological characteristics could potentially be shared with third parties to influence their future academic or career opportunities. To prevent such risks, the information retention period should be limited to the minimum necessary to achieve its purpose, and the data should be promptly deleted once the period expires. In addition, it is crucial to clearly define who is responsible for data management and assign responsibilities accordingly. For instance, when university researchers use data, clear agreements or contracts should specify the research purpose and scope of use to prevent its inappropriate use. However, when private companies manage healthtech, strict legal regulations and contracts are required to ensure that data are not exploited for commercial purposes that go beyond the intended scope of educational or health-related benefits. For instance, using student data to enhance algorithms that improve child protection and welfare may be considered legitimate use. However, repurposing the data for targeted advertising, user profiling for commercial products, or selling aggregated datasets to third parties should be strictly prohibited to prevent the misuse of sensitive student information.

Furthermore, access to the information collected should be restricted to a minimum number of relevant personnel. For example, only those directly supporting students, such as teachers and counselors, should be allowed to access the data. External individuals, including researchers, corporate staff, and members of the board of education, should be granted access only to anonymized data. The risk of identifying individual students can be minimized by anonymizing and encrypting the data.

When information collected through healthtech is linked to other data (eg, academic test scores or family background information), it is necessary to verify that the process and management system for linking these data are adequate. Although the linked data can potentially contribute to comprehensive support for individual students, improper handling may lead to privacy violations or reinforce bias. For example, there is a risk that a particular student may be unfairly labeled as “problematic.” To avoid such risks, it is essential that the data-linking process is conducted fairly and transparently, with clear definitions of the purposes for which the linked data will be used. By addressing these considerations, healthtech can be used to promote mental health while safeguarding students’ privacy and rights.

### Evaluation

When healthtech evaluates students’ mental health, it is possible that their responses may converge toward answers perceived as “favorable” or “likely to receive a good evaluation.” For example, when questions about stress or anxiety are included, students may conceal their true feelings and choose responses that they believe are more acceptable to evaluators, driven by the desire to present themselves in a better light [22]. Such convergence can undermine the recognition of individual differences and diverse values, instead encouraging self-expression that conforms to standardized criteria. In addition, this may result in genuine mental health issues being overlooked. To address this, it is crucial to include open-ended questions in addition to multiple-choice formats that provide children with the opportunity to express their feelings and circumstances in their own words.

Moreover, quantifying elements such as mental health and psychological traits—domains that are inherently multifaceted and context-dependent—could lead to the problem of “overquantification.” These areas are not easily reduced to simple numerical values, and quantification attempts may inadvertently reinforce misunderstandings or biases. For instance, the richness and diversity of students’ emotions and behaviors may be overlooked when they are reduced to scores or indices. In addition, when evaluation results are presented as numerical values, educators and parents may overrely on these figures, leading to rigid educational policies or support strategies. To prevent this, it is imperative to avoid excessive reliance on quantified results. Instead, establishing a system that combines these results with interpretations based on observations of daily behaviors and attitudes by teachers and parents can facilitate comprehensive evaluations.

Finally, concerns have been raised that the “individual optimization” recommended by certain systems—meaning the provision of tailored resources and interventions based on a student’s unique needs and characteristics—may become a “de facto standard,” that is, a widely adopted default practice not formally validated, thereby solidifying specific values as universal norms. For example, a risk-alert algorithm designed for one system may be adopted elsewhere without verifying its suitability in the new system. In such cases, the system’s recommendations can be widely adopted in educational settings, potentially narrowing the diverse possibilities for each student and hindering flexible educational approaches. To mitigate this risk, it is essential to design algorithm evaluation criteria that incorporate diverse perspectives and establish mechanisms for regularly reviewing optimization recommendations. In addition, it is important to ensure that teachers and parents retain the ability to make flexible judgments regarding the system’s recommendations.

### Use

The evaluation results provided by healthtech should be collected primarily to support students’ development and well-being. However, using these results as selection materials for admission or academic progress can help identify students who may require early individual support and prepare to provide appropriate assistance after enrollment. However, when the evaluation results are used as these selection criteria, students and their parents may become overly conscious of the evaluation, potentially distorting its original purpose of promoting growth and providing support. In addition, using evaluation results in the selection process may lead to the labeling of certain scores or traits, unfairly categorizing students as “unfit” or “problematic.” Furthermore, data related to mental health or developmental conditions are highly sensitive, and their use in selection processes can increase the risk of violating the privacy of students. Therefore, using the evaluation results from healthtech for selection purposes requires a thorough and careful discussion.

In screenings or interventions conducted through healthtech, students who report issues or difficulties are more likely to receive risk assessment and support, whereas those who fail to report such issues may be overlooked. For example, students with introverted personalities or difficulty expressing themselves may struggle to articulate their challenges, leading the system to incorrectly determine that no problems exist. Conversely, students who frequently report issues may attract excessive attention, potentially leading to disproportionate allocation of limited support resources. The determination of support should not rely solely on the frequency or severity of the reported issues. It is also necessary to consider how to support students who struggle with self-expression or face latent challenges by supplementing self-report assessments with observational data from teachers and parents, daily behavior records, and consideration of factors such as students’ age, sex, and other demographics.

The use of data collected through healthtech should also be discussed in terms of its application as anonymized data. Although the use of anonymized data has significant educational, academic, and social potential, schools may not actively promote their use, potentially amid concerns about the risk of reidentification of individuals and the perception among parents and students that anonymized data constitutes “surveillance” or “data exploitation.” To enable flexible use of anonymized information, it is essential to clearly define its purpose, establish robust data management systems, and ensure careful consideration and transparency. With these measures in place, anonymized information can be used to allow all stakeholders to share their benefits.

### Nature as Public Institutions

Topics such as privacy and consent, as discussed earlier, extend beyond ELSI concerns in educational settings and are also relevant in fields such as mental health and psychiatry. However, the nature of public institutions should also be considered, especially in light of the increasing implementation of healthtech in schools. For example, careful consideration of multiple perspectives regarding the financial burden of digital devices is necessary. With digital transformation advancing rapidly in the education sector, there is a need to develop a sustainable support model that goes beyond merely providing hardware. When allocating public funds, it is crucial to consider not only the initial investment but also long-term maintenance, update, and support costs. Comprehensive support schemes can include initiatives by local governments and educational committees, cost-sharing models in collaboration with corporations, and the establishment of social support funds. Concrete support measures are particularly important in ensuring that students from economically vulnerable households are not excluded. For example, strategies such as device-leasing systems, subsidies for low-income households, and shared-use systems developed by local communities and educational institutions should be actively explored.

In Japan, schools established based on the School Education Act are primarily eligible for financial support. Schools that fall under article 1 of this law, commonly referred to as “Article One Schools,” include elementary, junior high, high, and special support schools, which operate in accordance with the standards set by the MEXT. These standards encompass aspects such as curriculum, facilities, and teacher placement. Only schools that met these criteria were considered eligible for public financial support and subsidies. However, many alternative schools that do not meet these criteria—and therefore do not fall under article 1 of the School Education Act—are not eligible for public financial support. Consequently, providing healthtech-related subsidies to these schools presents complex challenges in terms of educational diversity and fairness. Incorporating multiple perspectives is crucial when considering subsidies for healthtech. First, there is a need to clarify the purpose and effects of these subsidies. A rigorous evaluation is necessary to determine the extent to which these measures contribute to specific goals, such as student health. Establishing guidelines, including ensuring transparency in the use of funds, periodic verification of effectiveness, and mandating performance reports, is essential.

Furthermore, careful consideration should be given to ensuring alignment with long-term educational policies. Establishing national evaluation and recommendation standards for mental health requires particular caution, considering that students’ mental health is closely related to individual dignity, privacy, and diversity during the developmental stages. Diverse perspectives must be considered when establishing national standards. First, objective evaluation indicators should be developed based on scientific evidence. Simultaneously, a flexible framework is essential for respecting individual diversity and preventing uniform assessments. Therefore, a comprehensive approach that considers the age, developmental stage, cultural background, and individual characteristics is critical. Moreover, mental health standards must not promote stigma or prejudice but foster understanding and support. Multilayered considerations are necessary, including interdisciplinary evaluations by experts, reflections on the perspectives of those directly affected and their families, ongoing research and standard updates, and strict information management to safeguard privacy.

### Relationships With Private Companies

As an issue related to public institutions, it is essential to carefully examine their relationships with private companies. Kucirkova [23] observed that educational technologies typically tend to overlook the principles of learning sciences while including distracting elements or manipulative designs that could reduce learning effectiveness and have an indirect negative impact on mental health. This issue appears to be a potential concern for healthtech as well. There are several other issues to be considered. For instance, if educational institutions do not fully understand the nature of healthtech or the changes made to it, they risk becoming overly dependent on service providers and compromising their autonomy. Private companies operate through multiple mechanisms. First, while determining technical specifications and functionalities, there is a risk that companies’ commercial logic may take precedence over the needs of educational settings. Furthermore, through ongoing updates and maintenance contracts, schools may become increasingly dependent on specific corporate ecosystems in the long term. In addition, during data collection and analysis, companies may monopolize critical insights regarding educational processes, thereby gaining substantial influence over de facto educational policy formation.

Another concern is that private companies’ values and evaluation criteria may infiltrate public education, raising serious concerns regarding the neutrality and public nature of education. Through healthtech, the commercial and ideological values of companies may inadvertently permeate students’ minds. Evaluation criteria in education should be based on pedagogical perspectives and holistic development of children and should not be distorted by the profit motives or specific values of private enterprises. Therefore, the process of introducing healthtech must involve the participation of educational scholars, ethics experts, and parental representatives to establish multilayered checks and balances to ensure that technology does not deviate from the essential purposes of education.

Moreover, the student data collected through healthtech includes highly sensitive and personal information, and the misuse of such data by private companies for purposes other than those originally intended has the potential to seriously violate students’ human rights. Therefore, strict legal regulations, transparent consent processes, and rigorous data management and protection mechanisms are essential. Specifically, this includes clearly limiting data use purposes, prohibiting third-party sharing, conducting regular audits, implementing comprehensive consent processes involving both students and their parents, and ensuring the right to data deletion.

## Conclusions

So far, we have emphasized that using healthtech in Japanese school education requires addressing ELSIs from eight key perspectives: (1) consent, (2) rights and privacy, (3) algorithms, (4) data collection and management, (5) evaluation, (6) use, (7) nature of public institutions, and (8) relationships with private companies. The applicability of informed consent and opt-out mechanisms has been discussed from the perspective of consent, focusing on the challenge of respecting the intentions of both parents and children. In terms of rights and privacy, guaranteeing the right to refuse evaluation and the right to data deletion has been emphasized, requiring systems to ensure these rights. Third-party auditing, error correction mechanisms, and fair and unbiased evaluation criteria are essential algorithms. For information collection and management, appropriate handling of sensitive data, limitations on storage periods, and prevention of unauthorized data use are critical challenges. In evaluation, considerations include the risk of evaluations suppressing diversity, the dangers of “overquantification,” and the potential fixation of individual optimization as a de facto standard. Use focuses on the appropriateness of using evaluation results for selection purposes, disparities in support between children who express difficulties and those who do not, and cautious consideration of anonymized data use. Furthermore, the public nature of schools necessitates multilayered oversight mechanisms to maintain neutrality and public accountability in education while integrating technology. Finally, relationships with private companies underscore the importance of ensuring transparency in preventing the influence of commercial values on education.

This project incorporated 52 issues spanning 8 topics based on Ed Tech 101 [17] (Table 1). Figure 1 provides a visual summary of the topics to be considered before and after implementation, as well as the appropriate institutions for these discussions. It is worth noting that many issues span more than 1 topic. For example, the issue “Will not schools and teachers emulate the methods of healthtech to voluntarily collect excessive personal information?” relates not only to information collection but also to consent, further rights, and privacy. This finding underscores the need to examine each issue from multiple perspectives. The table (Table 1; Multimedia Appendix 1) serves as a reference for diverse stakeholders in healthtech, including school teachers, boards of education, university researchers, and developers in private companies. To facilitate discussion, we have included explanations for technical terms such as “informed consent” and “assent,” as well as less-intuitive concepts such as “risk assessment.” Furthermore, as many of the issues are related to laws such as the Constitution, the Basic Act on Education, and the Act on the Protection of Personal Information, we have also provided references to relevant legislation. One relevant case is the Asahikawa Standardized Testing Case, where the court held that teachers’ freedom of education, while constitutionally protected, can be reasonably limited by public interest and institutional frameworks. This suggests that healthtech should be implemented within educational governance structures, not solely at individual discretion. Another relevant case concerned the disclosure of bullying survey responses, in which access was limited due to the risk of student reidentification based on handwriting. This highlights the need to address reidentification risks in healthtech design to protect student privacy, even when data are anonymized.

The introduction of healthtech brings new possibilities to educational settings [3,4]; however, it must be implemented in a fair and ethical manner. As a domestic example of a policy model for healthtech governance in education, we referred to the Education Data Utilization Roadmap [24] issued by Japan’s Digital Agency. This document outlines core principles, such as clarity of purpose, interoperability, and informed consent, all of which are highly relevant to the ELSI framework for implementing healthtech in schools. Notably, it emphasizes that data use should be designed not for administrative efficiency or vendor convenience but based on the best interests of children. This national-level policy serves as a concrete reference for aligning healthtech governance with ethical and educational priorities. Stakeholders involved in healthtech are encouraged to refer to this table, engage in thorough discussions tailored to their specific contexts, and base their practices on these deliberations.

To address the risks identified in this study, a phased approach is recommended. In the short term, concrete guidelines for obtaining consent should be developed, and efforts should be made to enhance the transparency of algorithmic decision-making. Pilot implementation of third-party audits could be introduced in selected educational settings. In the medium term, mechanisms for auditing and validating healthtech tools should be gradually established at the level of education boards or national authorities. Systems should also be put in place to collect feedback from users—including students, parents, and teachers—and to allow for correction requests. In the long term, policies promoting fairness and bias reduction should be integrated into educational guidelines. In addition, the sharing of international data privacy standards and best practices should be encouraged to support responsible and effective use of healthtech.

This study primarily focuses on Japan’s education system, which is characterized by a relatively centralized structure governed by national standards and policies. As such, the generalizability of the identified ELSI concerns and recommendations may vary in countries with decentralized or locally governed education systems. Further research is needed to explore how these considerations might differ in diverse educational contexts. However, the insights gained here provide valuable implications for discussions on the implementation of healthtech in educational settings worldwide. The ELSIs of healthtech—such as privacy, consent, and algorithmic transparency—are fundamental principles that can be applied to educational systems globally. However, its implementation must be adapted to each country’s legal framework and cultural contexts, particularly considering differences in public education policies and operational guidelines. For example, in the European Union, the General Data Protection Regulation establishes strict standards for data protection, including specific provisions for data processing in educational contexts. In the United States, the Family Educational Rights and Privacy Act governs the access to and privacy of student education records. These frameworks provide useful reference points for understanding how ELSI principles are operationalized in diverse legal systems and highlight the importance of adapting healthtech practices to meet international privacy and consent standards. Future research should further explore the applicability and challenges of healthtech across different educational systems through international comparisons, contributing to the development of more universal and practical guidelines.

## Appendix

Multimedia Appendix 1 Ethical, legal, and social issues on health technology in the educational context (English/Japanese).

## Abbreviations

DHC: Digital Health Contact
ELSIs: ethical, legal, and social issues
healthtech: health technology
MEXT: Ministry of Education, Culture, Sports, Science and Technology
