# Supplementary material for: Addressing the Ethical, Legal, and Social Issues of Healthtech in Education: Insights From Japan
Source: JMIR Form Res. 2025 Jul 18;9:e72781. doi: 10.2196/72781 (PMC12317288; doi:10.2196/72781)
Supplement: Multimedia Appendix 1 [file formative_v9i1e72781_app1.pdf]

**Multimedia Appendix 1. Ethical, legal, and social issues on health technology in the educational context**

| Number | Relevant laws and regulations | Issues | Consent | Rights & Privacy | Algorithms | Information acquisition & Management | Evaluation | Utilization | Public institutions | Private companies |
|---|---|---|---|---|---|---|---|---|---|---|
| When data collection and mental health assessments are conducted as a school activity, | | | | | | | | | | |
| 1 | Constitution, Article 26, Paragraph 1:"All people shall have the right to receive an equal education correspondent to their ability" | Is parental/guardian informed consent[1] required? If so, is the informed assent[2] of children/students also obtained? | ● | | | | | | | |
| 2 | Constitution, Article 26, Paragraph 1:"All people shall have the right to receive an equal education correspondent to their ability" | Does the information used in informed consent include risk assessment[3]? | ● | | | | | | | |
| 3 | Constitution, Article 26, Paragraph 1:"All people shall have the right to receive an equal education correspondent to their ability" | Even in the case where a parent (guardian) has not consented to data acquisition from a HealthTech service, are children/students notified of the details of parental consent/non-consent? | ● | | | | | ○ | | |
| 4 | Constitution, Article 26, Paragraph 1:"All people shall have the right to receive an equal education correspondent to their ability" | Should an “opt-out[4]” format be applied for consent? | ● | | | | | | | |
| 5 | | Do children or students view the facial features and attitudes of teachers and urge their parents or guardians to give their consent, or do they express support? | ● | | | | | | | |
| 6 | Constitution, Article 26, Paragraph 1:"All people shall have the right to receive an equal education correspondent to their ability" | Is there recognition of a right not to be evaluated by HealthTech? | | ● | | | ○ | | | |
| 7 | Constitution, Article 26, Paragraph 1:"All people shall have the right to receive an equal education correspondent to their ability" | To whom, to what extent, and how are the evaluation and reasons disclosed. | | ● | | | | ○ | | |
| 8 | ・Constitution, Article 26, Paragraph 1:"All people shall have the right to receive an equal education correspondent to their ability"<br>・Constitution, Article 13:"Personal rights" and "Right to privacy" | Is the so-called “right to be forgotten[5]” (e.g., the deletion of information collected by HealthTech) recognized? | | ● | | ○ | | | | |

| | | | | | | | | | | |
|---|---|---|---|---|---|---|---|---|---|---|
| 9 | Constitution, Article 26, Paragraph 1:"All people shall have the right to receive an equal education correspondent to their ability" | Issue requiring discussion: Do inferences regarding the internal mind (attitudes, emotions, etc.) of children/students constitute a failure to protect the freedom of thought and conscience of the concerned persons? | ○ | ● | ○ | | | | | |
| 10 | Basic Act on Education, Article 16:"Education must not be subject to improper controls" | Is this not considered an infringement of the teacher's right to "educational freedom[6]"? | | ● | | | | | | |
| 11 | Constitution, Article 13:"Personal rights" and "Right to privacy" | Does using expression or voice recognition to record the details of student information entail making sensitive information available to others, with a risk of violating privacy rights? | ○ | ● | ○ | | | | | |
| 12 | ・Constitution, Article 26, Paragraph 1:"All people shall have the right to receive an equal education correspondent to their ability"<br>・Characteristics of Japanese-style public education not necessarily grounded in law | Is third-party oversight (auditing, inspection) guaranteed for algorithms[7]? | | | ● | | | | | |
| 13 | Constitution, Article 26, Paragraph 1:"All people shall have the right to receive an equal education correspondent to their ability" | Do mechanisms exist for corrections where, due to inaccurate profiling, mistakes have occurred in the evaluation process? | | | ● | | | | | |
| 14 | Constitution, Article 26, Paragraph 1:"All people shall have the right to receive an equal education correspondent to their ability" | Can the system consider the need to ensure that children who deviate from the norm are not assessed unfavorably? | | | ● | | ○ | | | |
| 15 | Article 3 of the Educational Personnel Certification Act: 'Educational personnel must be those who have been granted a certificate in accordance with the provisions of this Act. | Is mental health expertise or third-party certification system required to develop assessment items and algorithms of HealthTech? If so, what are the criteria for determining it? | | | ● | | | | | |
| 16 | Constitution, Article 26, Paragraph 1:"All people shall have the right to receive an equal education correspondent to their ability" | Should sensitive personal information[8] about students with developmental disabilities and mental health history be acquired and used to screen and sort schools? | | ○ | ○ | ● | ○ | ○ | | |
| 17 | Constitution, Article 13:"Personal rights" and "Right to privacy" | Is the information management system regarding information accumulation and management as well as its linking with other information sufficient? | | | | ● | | | | |
| 18 | | Who, where, and how long should the information collected by HealthTech be stored? | | | | ● | | | | |
| 19 | Act on the Protection of Personal Information | How should requests for the cessation of use[9], etc., of collected data be treated? | | ○ | | ● | | | | |
| 20 | Act on the Protection of Personal Information | Will not schools and teachers emulate the methods of HealthTech to | ○ | ○ | | ● | | | | |

| | | | | | | | | | | |
|---|---|---|---|---|---|---|---|---|---|---|
| | | voluntarily collect excessive personal information? | | | | | | | | |
| 21 | Constitution, Article 26, Paragraph 1:"All people shall have the right to receive an equal education correspondent to their ability" | When an evaluation includes parameters other than just HealthTech assessment, how can it be guaranteed that factors indubitably related to prejudice/discrimination do not influence the evaluation? | | | | | ● | | | |
| 22 | Basic Act on Education, Article 16:"Education must not be subject to improper controls" | Does making health/mental condition “visible” promote hierarchization of students by grades? | | | | | ● | ○ | | |
| 23 | Basic Act on Education, Article 16:"Education must not be subject to improper controls" | Is there no danger of so-called “over-measurement” from quantifying things that are difficult to quantify? | | | ○ | | ● | | | |
| 24 | Constitution, Article 13:"Personal rights" and "Right to privacy" | Is there a risk that visualizing mental health assessments rather than academic evaluation provide a biased view of children? If visualization (with charts, etc.) focuses on mental health assessments rather than school life, is it not possible that more importance will be given to students’ abilities, leading to an over-bias on the importance of a person’s background? | | | | | ● | ○ | | |
| 25 | Basic Act on Education, Article 1:"Education must be provided with the aim of fully developing the individual character" | If such a face recognition system encourages a majority of students to adopt expression methods and emotional expressions that tend to result in good score, does this not hinder the diversity of self-expression? | | | | | ● | | | |
| 26 | Act on the Protection of Personal Information | How should one consider and treat cases where sensitive individual information is used to infer, via profiling, that the concerned individual requires special care or consideration? | | | | | ● | ○ | | |
| 27 | | What are the grounds for making all problems identical for everyone? Is it necessary to modify the items and assessment methods according to regional or age differences? | | | ○ | | ● | | | |
| 28 | | The use of technologies is expected to increase equality in evaluation of interests, motivation, and attitudes compared with the subjective evaluation of human teachers. However, will objective quantification (scoring) of such things as developmental disorders and/or mental illness result in such conditions being treated in a fixed, inflexible way? | | | ○ | | ● | ○ | | |
| 29 | | With the accumulation of recordings of a child/student’s past problematic behavior, even if that child shows growth and development (maturation) over time, will that child/student not be | | | | | ● | | | |

| | | | | | | | | | | |
|---|---|---|---|---|---|---|---|---|---|---|
| | | “stuck with” that reputation, etc., making appropriate evaluations in the future unlikely? | | | | | | | | |
| 30 | | Can mental health history and abilities be linked to personality evaluation? | | | | | ● | | | |
| 31 | | When the “optimal” value for “individual optimization[10]” recommended by a system is not proven with evidence, is there not a risk that the “individual optimization” as designated by the system in its first introduction and use becomes a type of de facto standard[11], locking in those values as “standards?” | | | | | ● | | | |
| 32 | Constitution, Article 26, Paragraph 1:"All people shall have the right to receive an equal education correspondent to their ability" | Are there assurances that the results of evaluations performed to promote the development of a child/student are not used in selection screening? | | | | | ○ | ● | | |
| 33 | Basic Act on Education, Article 16:"Education must not be subject to improper controls" | It is acceptable if HealthTech is used by a teacher as a support tool, but if its influence surpasses that of human judgment, would that lead to “inappropriate control” by HealthTech? | | | | | | ● | | |
| 34 | Constitution, Article 89:"Expenditures of public money, and limits on its usage (appropriation)" | Is HealthTech truly used for educational purposes, and not just nominally so? | | | | | | ● | | |
| 35 | Constitution, Article 14:"Equality under the law" and "Prohibition of discrimination" | Will classifications (for determining members of specific school classes, etc.) based on differences in assessment by HealthTech ultimately not lead to discrimination? | | | | | ○ | ● | | |
| 36 | Constitution, Article 14:"Equality under the law" and "Prohibition of discrimination" | If students' posture and attitude are judged based on recognition accuracy (i.e., some faces tend to be recognized by face recognition as actively claiming and others as expressionless), does this constitute discrimination? | | | | | ○ | ● | | |
| 37 | Every Article and item (paragraph) of the Local Public Service Act stipulated by this Act." | Should educational results from HealthTech be recognized as the educational performance of teachers? | | | | | ○ | ● | | |
| 38 | Every Article and item (paragraph) of the Local Public Service Act stipulated by this Act." | Is it appropriate to use data obtained through facial recognition[12], such as a teacher's level of concentration and facial expressions, as criteria for teacher evaluation? | | | | | ○ | ● | | |
| 39 | Act on the Protection of Personal Information | How should the usage of anonymously processed information[13] be treated? | | | | | | ● | | |
| 40 | | Is it necessary to add HealthTech functions that prioritize student guidance elements, such as those that help improve postures or those that boost students' self-worth by | | | ○ | | | ● | | |

| | | | | | | | | | | |
|---|---|---|---|---|---|---|---|---|---|---|
| | | encouraging them to modify their behavior? | | | | | | | | |
| 41 | | Will the teacher's duties not be taken over in those areas where HealthTech can serve as a substitute or alternate performer? | | | | | | ● | | |
| 42 | Constitution, Article 26, Paragraph 1:"Right to receive education "Paragraph 2: "Compulsory education shall be free" | If there is evidence of educational benefits, is the national government obliged to introduce an educational environment using HealthTech? | | | | | | | ● | |
| 43 | Constitution, Article 26, Paragraph 1:"Right to receive education "Paragraph 2: "Compulsory education shall be free" | In the case of digital terminals (tablets, LAN extension inside the student's home etc.), will the cost of purchasing related educational materials be privately or publicly funded? | | | | | | | ● | |
| 44 | Basic Act on Education, Article 16:"Education must not be subject to improper controls" | If, for example, a system is established where subsidies and other payment standards vary depending on whether or not a certain school has introduced HealthTech promoted by the Ministry of Education, Culture, Sports, Science and Technology, could this lead to the penetration of the government's message to children via public education? | | | | | | | ● | |
| 45 | Constitution, Article 89:"Expenditures of public money, and limits on its usage (appropriation)" | Can assistance monies be paid to an educational institution operated by an HealthTech enterprise (i.e., an enterprise not designated under Article 1 of the School Education Act[14,15])? | | | | | | | ● | |
| 46 | Basic Act on Education, Article 1:"Education must be provided with the aim of fully developing the individual character" | Is it appropriate for the national government to set uniform evaluation standards? | | | | | | | ● | |
| 47 | Characteristics of Japanese-style public education not necessarily grounded in law | Does the mass introduction of HealthTech services entail expenditures of public monies for private businesses? | | | | | | | ● | ○ |
| 48 | Characteristics of Japanese-style public education not necessarily grounded in law | Will the use of HealthTech to teach about mental health be considered health education content in school curricula? | | | | | | ○ | ● | |
| 49 | Basic Act on Education, Article 16:"Education must not be subject to improper controls" | Is it suitable for HealthTech provided by private enterprises to be incorporated into educational contents and/or methods? | | | | | | | | ● |
| 50 | Constitution, Article 89: "Expenditures of public money, and limits on its usage (appropriation)" | If HealthTech is introduced without sufficient knowledge of information and communications technology (ICT)[16], schools may be unable to fully understand its content and ongoing changes, with the result that they | ○ | | | | ○ | ○ | | ● |

| | | | | | | | | | | |
|---|---|---|---|---|---|---|---|---|---|---|
| | | merely follow what the tech provider instructs. | | | | | | | | |
| 51 | ・Constitution, Article 13:"Personal rights" and "Right to privacy"<br>・Basic Act on Education, Article 1:"Education must be provided with the aim of fully developing the individual character" | Will the value systems and expressions of private companies be linked with the evaluation standards for children/students that are used in public education? | | | | | | | | ● |
| 52 | School Education Act, Article 2: "Schools shall be established only by the national government [ . . . ], local governments [ . . . ], and school corporations specified by Article 3 of the Private Schools Act [ . . . ]" | If a stockholding company, not an incorporated educational institution, possesses multifaceted data of students, is there a risk that the data may be used beyond education-related purposes? | | | | ○ | | | | ● |

●: Primary Categories of Consideration. ○: Supporting Categories of Consideration.

[1] Informed consent: providing consent after having been fully informed and satisfied about the purpose, risks, benefits, and alternatives of the data collection or intervention.

[2] Informed assent: In addition to parental consent (informed consent) for minors and others, the child’s consent should be obtained after he or she understands and accepts the purpose, risks, benefits, and alternatives to data collection or intervention.

[3] Risk assessment: Assessing potential risks and harms that may arise from implementing HealthTech in advance, and considering measures to mitigate those risks.

[4] Opt-out: A process in which individuals, in a situation where they are automatically included in a specific service or data processing, request to be excluded based on personal decision.

[5] Right to be forgotten: The right of an individual to request the deletion of their personal information stored by an organization or service provider when they ask for it to be erased.

[6] Freedom of educational policy: Schools and teachers have the right to freely decide on their educational policy. A famous court case is the Asahikawa School Achievement Test. The “Asahikawa Gakute Judgment” in the 1950s is a leading case involving the freedom of education of school teachers (here, a teacher who opposed the National Achievement Test (or “Gakute”) was charged with obstructing the execution of public affairs).

[7] Algorithm: A set of specific methods, processes, or rules established to address a particular issue or conduct analysis.

[8] Sensitive personal information: Personal information that requires particularly careful handling, such as health status, disability, nationality, criminal history, or beliefs.

9 Request for suspension of use: The right of an individual to request the suspension or deletion of personal information when its use is illegal or inappropriate.

10 Individual optimization: Providing optimal measures and resources based on an individual's needs and characteristics.
11 De facto standards: Technologies or norms recognized as the default through widespread adoption and proven track record, without undergoing formal standardization.
12 Face recognition: Analyzing facial features and using it to identify the individual.
13 Anonymously Processed Information: Data generated by removing or replacing necessary identifying elements from personal information to ensure that specific individuals cannot be identified.
Fourteen schools falling under School Education Act 1: Educational institutions (so-called " schools) established under Article 1 of the "School Education Act." Specifically, this refers to kindergarten, elementary, junior high, compulsory education, high, secondary education, and special needs education schools, universities, and college of technology (KOSEN).
Fifteen schools that do not fall under School Education Act 1: Educational institutions not positioned under Article 1 of the "School Education Act." Specifically, they are international, driving, and preparatory schools.
16 ICT (Information and Communication Technology): This term collectively refers to technologies and tools related to information processing and communication, such as computers, the Internet, smartphones, and cloud services.

**Multimedia Appendix 2. 教育現場での HealthTech に関する倫理的・法的・社会的課題（ELSI）**

| 番号 | 法令 | | 同意 | 権利/プライバシー | アルゴリズム | 情報取得/管理 | 評価 | 活用 | 公的機関 | 民間企業等 |
|---|---|---|---|---|---|---|---|---|---|---|
| HealthTech によるデータ取得やメンタルヘルス評価が学校での活動として実施される場合、 | | | | | | | | | | |
| 1 | 憲法 26 条 1 項「能力に応じて、ひとしく教育を受ける権利」 | 保護者へのインフォームド・コンセント(同意)[1] は必要か。必要な場合、子どもへのインフォームド・アセント(賛意)[2] もとるか。 | ● | | | | | | | |
| 2 | 憲法 26 条 1 項「能力に応じて、ひとしく教育を受ける権利」 | インフォームド・コンセントにリスクアセスメント [3] に関する内容を含めるか。 | ● | | | | | | | |
| 3 | 憲法 26 条 1 項「能力に応じて、ひとしく教育を受ける権利」 | HealthTech によるデータ取得に保護者が同意しなかった場合でも、その子どもがその HealthTech を活用できるようにしてよいか。その場合、保護者の同意/不同意内容を子どもに通知するか。 | ● | | | | | ○ | | |
| 4 | 憲法 26 条 1 項「能力に応じて、ひとしく教育を受ける権利」 | 同意(取得) をオプトアウト [4] 方式にしてよいか。 | ● | | | | | | | |
| 5 | | 子どもや保護者が教師の顔色を見て、同意/不同意を示すことはないか。 | ● | | | | | | | |
| 6 | 憲法 26 条 1 項「能力に応じて、ひとしく教育を受ける権利」 | 子どもには HealthTech による評価を受けない権利が認められるか。 | | ● | | | ○ | | | |
| 7 | 憲法 26 条 1 項「能力に応じて、ひとしく教育を受ける権利」 | 評価結果やその理由は、誰にどこまでどのように開示されるか。 | | ● | | | | ○ | | |
| 8 | ・憲法 26 条 1 項「能力に応じて、ひとしく教育を受ける権利」<br>・憲法 13 条「人格権」「プライバシー権」 | HealthTech によって取得された情報の削除等、すなわち「忘れられる権利 [5]」は認められるか。 | | ● | | ○ | | | | |

| | | | | | | | | | | |
|---|---|---|---|---|---|---|---|---|---|---|
| 9 | 憲法 26 条 1 項「能力に応じて、ひとしく教育を受ける権利」 | HealthTech によって得られたデータから子ども個々人が外部に表出することを望まない、彼らの内面（感情など）の部分を可視化していいか。 | ○ | ● | ○ | | | | | |
| 10 | 教育基本法 16 条「教育は不当な支配に服することなく」 | HealthTech による評価を一律に導入することによって、教師の教育方針の自由[6]を奪うことにはならないか。 | | ● | | | | | | |
| 11 | 憲法 13 条「人格権」「プライバシー権」 | 子ども個々人が話したくない知られたくないと思っているセンシティブな情報を、表情や音声等からシステムが察知できるとすれば、プライバシーを侵害するおそれがないか。 | ○ | ● | ○ | | | | | |
| 12 | ・憲法 26 条 1 項「能力に応じて、ひとしく教育を受ける権利」<br>・法律上の根拠が必ずしも明確でない日本型公教育の特徴 | アルゴリズムについて第三者による監査可能性は担保されているか。 | | | ● | | | | | |
| 13 | 憲法 26 条 1 項「能力に応じて、ひとしく教育を受ける権利」 | 不正確な分析結果によって、間違った評価を行った場合に修正される仕組みはあるか。 | | | ● | | | | | |
| 14 | 憲法 26 条 1 項「能力に応じて、ひとしく教育を受ける権利」 | 標準から外れた子どもに不利な評価が生じないような配慮をシステムに組み込めるか。 | | | ● | | ○ | | | |
| 15 | 教育職員免許法 3 条「教育職員は、この法律により授与する各相当の免許状を有する者でなければならない。」 | HealthTech の評価項目やアルゴリズムの作成者にメンタルヘルスの専門的知識が必要か。必要な場合、専門的知識があると判断する基準は何か。 | | | ● | | | | | |
| 16 | 憲法 26 条 1 項「能力に応じて、ひとしく教育を受ける権利」 | HealthTech によって、発達障害や精神疾患等の要配慮個人情報[8]を取得してよいか。取得できる場合、それを学級編成等に使ってよいか。 | | ○ | ○ | ● | ○ | ○ | | |
| 17 | 憲法 13 条「人格権」「プライバシー権」 | HealthTech によって取得した情報の管理及び他情報との連結に関する情報管理体制は十分か。 | | | | ● | | | | |
| 18 | | HealthTech によって取得した情報は、だれが、どこで、どのくらい保存するのか。 | | | | ● | | | | |

| | | | | | | | | | | |
|---|---|---|---|---|---|---|---|---|---|---|
| 19 | 個人情報保護法 | 過去に取得された情報に対する利用停止等の請求[9]を誰がどのように扱うか。 | | ○ | | ● | | | | |
| 20 | 個人情報保護法 | 学校・教員が HealthTech の手法を参照して、自発的かつ過剰に子どもの個人情報をとることはないか。 | ○ | ○ | | ● | | | | |
| 21 | 憲法 26 条 1 項「能力に応じて、ひとしく教育を受ける権利」 | メンタルヘルス以外の情報まで含めた評価が行われる場合、その評価が差別につながらないことをどのように保証するか。 | | | | | ● | | | |
| 22 | 教育基本法 16 条「教育は不当な支配に服することなく」 | メンタルヘルスの可視化が子どもの序列化を生まないか。 | | | | | ● | ○ | | |
| 23 | 教育基本法 16 条「教育は不当な支配に服することなく」 | メンタルヘルスという数値化しづらいものを数値化する、いわゆる「測りすぎ」の弊害はないか。 | | | ○ | | ● | | | |
| 24 | 憲法 13 条「人格権」「プライバシー権」 | 可視化できるものが学業よりもメンタルヘルス評価に集中すると、子どもを見る視点がメンタルヘルスに偏るのではないか。 | | | | | ● | ○ | | |
| 25 | 教育基本法 1 条「人格の完成」 | メンタルヘルスが評価されることで、良い評価を得やすい回答に収れんされ、多様性が阻害されてしまうのではないか。 | | | | | ● | | | |
| 26 | 個人情報保護法 | 取得した情報から推知されるメンタルヘルスの状態を誰がどのように判断し、どう扱うか。 | | | | | ● | ○ | | |
| 27 | 該当法令なし | 地域や年齢によって、評価の項目や方法を変える必要はあるか。 | | | ○ | | ● | | | |
| 28 | 該当法令なし | HealthTech を用いてメンタルヘルスを数値化することで客観性は増すが、発達障害や精神疾患等もまた客観的に数値化され、固定的に扱われてしまわないか。 | | | ○ | | ● | ○ | | |
| 29 | 該当法令なし | 過去の HealthTech によって取得された情報にとらわれると、例えそこから変化が見られても、その子どもの変化を適切に評価できなくなるのではないか。 | | | | | ● | | | |
| 30 | 該当法令なし | 過去に取得された情報が子どもの人格評価と結びつけられないか。 | | | | | ● | | | |

| | | | | | | | | | | |
|---|---|---|---|---|---|---|---|---|---|---|
| 31 | 該当法令なし | 特定のシステムによって推奨された個別最適[10]が「最適」であることの証明がなされないまま、デファクトスタンダード（事実上の標準）[11]となり価値が固定化されないか。 | | | | | ● | | | |
| 32 | 憲法 26 条 1 項「能力に応じて、ひとしく教育を受ける権利」 | 子どもの発達を促すために行ってきた評価結果を、入試等の選抜資料として使ってよいか。 | | | | | ○ | ● | | |
| 33 | 教育基本法 16 条「教育は不当な支配に服することなく」 | HealthTech をメンタルヘルスの判断補助として用いるならよいが、人間による判断を上回る影響力を持たせないようにするにはどうしたらよいか。 | | | | | | ● | | |
| 34 | 憲法 89 条「公の財産の支出又は利用の制限」 | 導入している HealthTech は、本当に教育目的にかなっているか、また形骸化していないか。 | | | | | | ● | | |
| 35 | 憲法 14 条「法の下の平等」「差別の禁止」 | HealthTech の結果による学級編成が結果的に差別につながらないか。 | | | | | ○ | ● | | |
| 36 | 憲法 14 条「法の下の平等」「差別の禁止」 | HealthTech で不調を訴える子どもほどリスクアセスメント[3]や介入を受けやすくなるが、一方で不調を訴えない子どもがそれらを受けにくくならないか。 | | | | | ○ | ● | | |
| 37 | 地方公務員法の各条項 | HealthTech を用いて計測された子どものメンタルヘルスの変化を教員の教育効果として認めてよいか。 | | | | | ○ | ● | | |
| 38 | 地方公務員法の各条項 | 顔認識[12]等により取得した、教員の集中度や表情等を教員評価の対象としてよいか。 | | | | | ○ | ● | | |
| 39 | 個人情報保護法 | 匿名加工情報[13]をどのように利活用するのか。 | | | | | | ● | | |
| 40 | 該当法令なし | 子どもの行動変容を促すような HealthTech 機能の付加は必要か。 | | | ○ | | | ● | | |
| 41 | 該当法令なし | HealthTech を用いた評価が、教員の健康観察や保健教育に関する業務を奪うことにならないか。 | | | | | | ● | | |
| 42 | 憲法 26 条 1 項「教育を受ける権利」2 項「義務教育は、これを無償とする」 | HealthTech にメンタルヘルスの低下予防効果のエビデンスがあれば、国家は HealthTech を用いた教育環境を整備する義務を負うか。 | | | | | | | ● | |

| | | | | | | | | | | |
|---|---|---|---|---|---|---|---|---|---|---|
| 43 | 憲法 26 条 1 項「教育を受ける権利」2 項「義務教育は、これを無償とする」 | HealthTech に用いるデジタル端末にかかる費用（端末購入やメンテナンス料、家庭における LAN の設置・使用料）は、誰が負担するか。 | | | | | | | ● | |
| 44 | 教育基本法 16 条「教育は不当な支配に服することなく」 | HealthTech に公的資金が用いられる場合、政府による HealthTech を推奨するようなメッセージを浸透させることはないか。 | | | | | | | ● | |
| 45 | 憲法 89 条「公の財産の支出又は利用の制限」 | 学校教育法第 1 条に該当しない学校[14,15]に HealthTech のための補助金を支出してよいか。 | | | | | | | ● | |
| 46 | 教育基本法 1 条「人格の完成」 | メンタルヘルスに対する評価基準や推奨基準を国家として提示してよいか。 | | | | | | | ● | |
| 47 | 法律上の根拠が必ずしも明確でない日本型公教育の特徴 | HealthTech サービスの大量導入は民間企業への公金支出にあたらないか。 | | | | | | | ● | ○ |
| 48 | 法律上の根拠が必ずしも明確でない日本型公教育の特徴 | HealthTech を用いて、メンタルヘルスについて子どもが知ることは学校教育における保健教育の内容として認められるか。 | | | | | | ○ | ● | |
| 49 | 教育基本法 16 条「教育は不当な支配に服することなく」 | 民間企業等が HealthTech を通して、教育の内容や方法に関与してよいか。 | | | | | | | | ● |
| 50 | 憲法 89 条「公の財産の支出又は利用の制限」 | ICT[16]についてよくわからないまま導入を図ると学校側がその内容や変更点などが掌握できず、事業者のいいなりになってしまわないか。 | ○ | | | | ○ | ○ | | ● |
| 51 | ・憲法 13 条「人格権」「プライバシー権」<br>・教育基本法 1 条「人格の完成」 | Health Tech の導入によって民間企業等の価値観や表現が、子どもに浸透させられたり、公教育の中で用いられる評価基準へ結びつけられたりしないか。 | | | | | | | | ● |
| 52 | 学校教育法 2 条「学校は、国…、地方公共団体…及び私立学校法…第三条に規定する学校法人…のみが、これを設置することができる。」 | 民間企業等が、HealthTech で取得した子どものデータを、教育利用以外へ転用するおそれがないか。 | | | | ○ | | | | ● |

注）●：主要検討カテゴリー。○：副検討カテゴリー。[1] インフォームドコンセント：データ収集や介入の目的、リスク、利益、代替手段などについて十分な説明を受け、納得した上で同意をすること。[2] インフォームドアセント：未成年者などの場合に、保護者が同意すること（インフォームドコンセント）に加え、子ども自身が、データ収集や介入の目的、リスク、利益、代替手段について理解し、納得した上で本人の同意を得ること。[3] リスクアセスメント：HealthTech を行うことによって生じる可能性のある危険性や有害性を事前に査定すること。また、そのリスクを低減するための対策を検討すること。[4] オプトアウト：個人が特定のサービスやデータ処理に自動的に含まれる状況において、本人が自らの意思でそのサービスやデータ処理から除外を求める手続き。[5] 忘れられる権利：組織やサービス提供者によって保存されている個人情報を、その個人情報の本人が「消してほしい」と要求した場合に削除される権利のこと。[6] 教育方針の自由：学校や教員が教育方針を自由に決められる権利のこと。有名な裁判として「旭川学力テスト事件」がある ※旭川学力事件：1950 年代、旭川で行われた学力テストを通じ、地方自治体が学校の教育方針に介入しすぎることによって、学校や教員の教育方針の自由が侵害されるのではないか、という事件。[7] アルゴリズム：特定の問題解決や分析を行うために定められた一定の手段やプロセス、ルールのこと。[8] 要配慮個人情報：特に慎重な取り扱いが求められる個人情報（例：健康状態、障害、国籍、犯罪的、信条など）のこと。[9] 利用停止等の請求：個人情報の利用に対して、違法または不適切である場合に、本人がその情報の利用停止や情報削除を求める権利のこと。[10] 個別最適：個人のニーズや特性に基づいて、最適な方策やリソースを提供すること。[11] デファクトスタンダード：正式な規格化を経ずに、普及や実績によって標準として認識された技術、規格。[12] 顔認識：個人の顔の特徴を解析し、これを用いて個人を識別すること。[13] 匿名加工情報：特定の個人を識別できないように、個人情報から必要な識別要素を除去または置き替えることで生成されたデータのこと。[14] 学校教育法第 1 条に該当する学校：「学校教育法」第 1 条に基づき設立される教育機関（いわゆる「学校」）のこと。一条校とも呼ばれる。具体的には、幼稚園、小学校、中学校、義務教育学校、高等学校、中等教育学校、特別支援学校、大学及び高等専門学校を指す。[15] 学校教育法第 1 条に該当しない学校：「学校教育法」第 1 条に位置付けられていない教育機関のこと。非一条校とも呼ばれる。具体的には、インターナショナルスクール、自動車学校、予備校など。[16] ICT（情報通信技術）：コンピュータやインターネット、スマートフォン、クラウドサービスなど、情報の処理や通信に関連する技術やツール全般を総称する用語。
